# Effect of the Cationic Head Group on Cationic Surfactant-Based Surfactant Mediated Gelation (SMG)

**DOI:** 10.3390/ijms21218046

**Published:** 2020-10-28

**Authors:** Kenji Aramaki, Eriko Takimoto, Takumi Yamaguchi

**Affiliations:** Graduate School of Environment and Information Sciences, Yokohama National University, Yokohama 240-8501, Japan; takimoto-eriko-rx@ynu.jp (E.T.); yamaguchi-takumi-ks@ynu.jp (T.Y.)

**Keywords:** surfactant-mediated gelation, hydrogel, organogelator, surfactant, orthogonal system, self-assembly

## Abstract

The surfactant-mediated gelation (SMG) method allows us to formulate hydrogels using a water-insoluble organogelator. In this study, we formulated hydrogels using three cationic surfactants, hexadecyltrimethylammonium bromide (CTAB), hexadecyltrimethylammonium chloride (CTAC), and hexadecylpyridinium chloride (CPC)] and an organogelator (12-hydroxyoctadecanoic acid (12-HOA), and studied their structures and mechanical properties. A fiber-like structure similar to that found in the 12-HOA-based organogels was observed by optical microscopy. Small- and wide-angle X-ray scattering profiles showed Bragg peaks derived from the long- and short-spacing of the crystalline structures in the gel fibers and a correlation peak from the surfactant micelles in the small-angle region. Furthermore, the formation of micelles in the hydrogels was confirmed by UV-vis spectroscopic measurements of the gel samples in the presence of Rhodamine 6G. We concluded that the hydrogels prepared by the SMG method in the present systems are orthogonal molecular assembled systems in which two different molecular assembled structures coexist. Among the three surfactant systems, the CTAB system presented the lowest critical gelation concentration and highest sol-gel transition temperature and viscoelasticity. These differences in gel fiber formation and gel properties were discussed from the viewpoint of the degree of solubilization of the gelator molecules in micelles coexisting with gel fibers and diffusion of the gelator molecules in the gel formation process.

## 1. Introduction

A low-molecular-weight gelator (LMG) forms micron-sized fiber-like molecular aggregates by crystallization from the solution state. Intertwined fibers, namely self-assembled fibrillar networks (SAFiNs), hold large amounts of solvent to form gels [1,2]. 12-Hydroxyoctadecanoic acid (12-HOA), a well-known LMG, is an organogelator that gels when dissolved in various organic solvents at high temperature and subsequently cooled. In the 12-HOA fiber-like molecular assembly, hydrogen bonds tightly bind the molecules to form a lamellar-stacked bilayer membrane [3,4,5,6,7]. Additionally, the fibers formed by 12-HOA molecules with high optical purity are helical with constant pitch. The cross-sectional shape of the fibrous molecular assembly depends on the type of solvent and gelator concentration [8,9,10]. In turn, the minimum gelation concentration and sol-gel transition temperature depend on the polarity of the solvent [10,11,12,13] and elastic gel rheological properties [10,11,14,15,16].

An LMG that gels organic solvents (organogelator) is generally unable to form hydrogels. A common route for gelation with an LMG is to dissolve it in a solvent at high temperature and subsequently cool the solution. In this case, the prerequisite for defining an LMG as an organogelator or hydrogelator is its solubility in a solvent at high temperature. For example, 12-HOA cannot be dissolved in water and, therefore, it cannot form hydrogels. However, if the organogelator can be dissolved in water by any means, a hydrogel is formed. In the case of 12-HOA, the water solubility can be increased, and hydrogels are formed by several approaches using the properties of the carboxyl group, such as pH adjustment, conversion to the metal salt, and formation of an ion complex with alkylamine [17,18,19,20,21]. Another approach, the physicochemical approach, utilizes the solubilization function of the surfactants and does not depend on the specific chemical properties of the gelators. A bicontinuous microemulsion (BME) and lamellar liquid crystal (L_α_) phase formed by a water/surfactant/oil system can solubilize 12-HOA at high temperature, and the gels are obtained by cooling [22,23,24,25,26,27,28]. In this case, the afforded gel is not purely a hydrogel because it contains micro-oil domains of BME or L_α_. Later, hydrogels have also been obtained by solubilizing 12-HOA in the oil-free L_α_ phase formed in a binary water/surfactant system [15,29]. Furthermore, we attempted hydrogel formation by adding 12-HOA to aqueous surfactant micellar solutions and successfully demonstrated hydrogel formation by solubilizing 12-HOA in a micellar solution of hexadecyltrimethylammonium bromide (CTAB) and the formation of a CTAB worm-like micellar solution in the presence of sodium salicylate. This physicochemical route for obtaining a hydrogel by an organogelator was named surfactant-mediated gelation (SMG) [16]. Hydrogels formed by the SMG method are (or approach) orthogonal systems in which the surfactant and supramolecular aggregates of the gelator coexist [30,31]. In biological cell structures, the orthogonal system comprises a lipid bilayer membrane with high flexibility and self-healing properties that coexist with a cytoskeleton of high mechanical strength. The hydrogels obtained by the SMG method have a similar complex mixture of soft and hard structures. Therefore, research on the SMG method could contribute toward the development of biomimetic technology and the greater sophistication of the science of molecular assemblies. Additionally, the discovery of such a novel approach not only expands the types of solvents that can be gelled for a given LMG but also provides opportunity for a deeper understanding of the SAFiN formation and gel stabilization mechanisms. When organogels are formulated by 12-HOA, a simple crystal growth process occurs, namely crystal nuclei and fiber growth occur from any places in a 12-HOA simple solution of a low polar solvent. However, during the SMG gelation, generation of crystal nuclei occurs from nanometer-sized limited spaces inside micelles. In addition, gelator molecules should be supplied to the crystal nuclei from other micelles. Therefore, the gel fiber evolution inevitably depends on the size and diffusivity of the micelle. To understand this point, we investigated the structures and properties of 12-HOA-based hydrogels formed by the SMG method with three different cationic surfactants in this study. The cationic surfactants employed in this study have different head group structures, which should affect gel fiber formation because the surfactant head group influence the micelle size and diffusivity in water.

## 2. Results

### 2.1. Gelation Tests

In the three-component system comprising a cationic surfactant, 12-HOA, and water, the surfactant concentration was fixed at 0.15 M. The states (gel or sol) of the samples of different 12-HOA concentrations (C_12-HOA_) were determined by the inverted test tube method (Section 4.3), and the results are summarized in Table 1. In the system with CTAB as the surfactant (hereafter referred to as “the CTAB system”), gelation was only observed above 1.0 wt.%, and the sample with C_12-HOA_ = 0.75 wt.% could not be gelled. Therefore, the critical gelation concentration (cgc) that enables sufficient formation of SAFiNs of the 12-HOA fiber-like aggregate to hold all the solvent in the system was within the C_12-HOA_ concentration range 0.75–1.0 wt.%. Hydrogels were also obtained in the CTAC and CPC systems with cgc values within the C_12-HOA_ concentration ranges 1.5–1.75 wt.% and 1.75–2.0 wt.%, respectively. Thus, the CTAB system was deemed the most efficient system for gelation. Sample photos at different 12-HOA concentrations (C_12-HOA_) are shown in Figure S1. The transparency of the hydrogels is translucent or turbid. The hydrogels in the CTAC system seems slightly higher transparency although the reason for this difference is not discovered at this moment.

### 2.2. Confirmation of the Hydrogel Structure and Orthogonality

**Figure 1:**
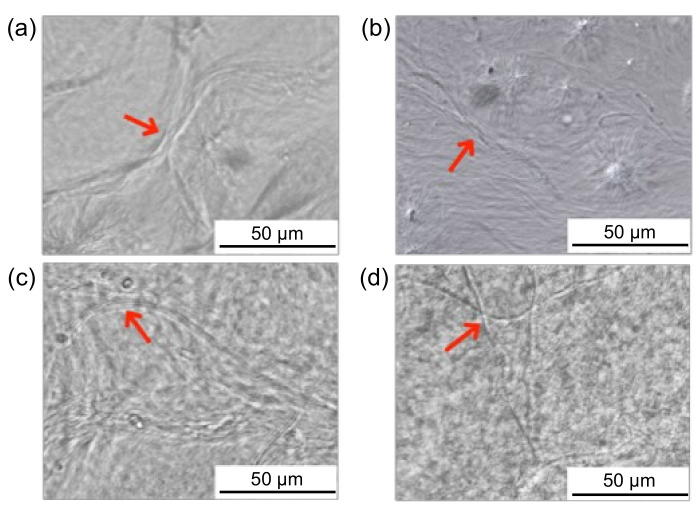
Optical micrographs of the hydrogels (**a**–**c**) and organogel (**d**): (**a**) hexadecyltrimethylammonium bromide (CTAB), (**b**) hexadecyltrimethylammonium chloride (CTAC), (**c**) hexadecylpyridinium chloride CPC, and (**d**) binary n-decane/12-hydroxyoctadecanoic acid (12-HOA) systems (concentration *C*_12-HOA_ = 3.0 wt.%). The red arrows indicate the aggregated fibers forming thicker fibers.

The hydrogels of the three different surfactant systems and the organogel (solvent: n-decane; *C*_12-HOA_ = 3.0 wt.% for the hydrogels and organogel) were observed under an optical microscope, and the results are shown in Figure 1. In all the hydrogels, fibers with micrometer-sized widths were observed, which were aggregated in several places (indicated by the red arrows) to form thicker fibers. The fibers were entangled in some places or branched to form a random network. A similar random fiber-like texture was formed in the organogel.

**Figure 2:**
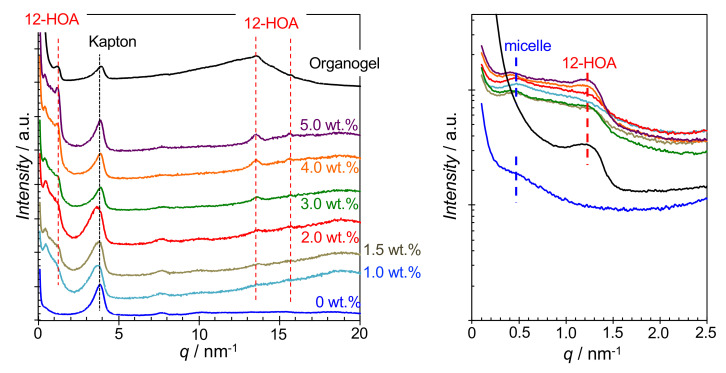
Small- and wide-angle X-ray scattering (SWAXS) spectra of the hydrogels in the hexadecyltrimethylammonium bromide (CTAB) system at different 12-hydroxyoctadecanoic acid concentrations (*C*_12-HOA_ = 1.0–5.0 wt.%), non-gelled CTAB micellar solutions (*C*_12-HOA_ = 0 wt.%), and organogel (*C*_12-HOA_ = 3.0 wt.%, in n-decane) at 25 °C. The small angle peaks at *q* ~4 nm^−^^1^ are from Kapton windows.

The small- and wide-angle X-ray scattering (SWAXS) results of the hydrogels formed in the CTAB system at 25 °C are shown in Figure 2. For comparison, SWAXS measurements were also performed on the organogel in the binary 12-HOA/n-decane system (*C*_12-HOA_ = 3.0 wt.%) and the results are also shown. Small-angle X-ray scattering (SAXS) of the micellar solution (*C*_12-HOA_ = 0 wt.%) displayed a shoulder in the range *q* = 0.3–0.8 nm^−1^, which is attributed to the interference X-ray scatterred from randomly distributed micelles. A broad peak at the same position was also observed in the hydrogel samples, suggesting that micelles also exist in the hydrogels. In the organogel small-angle region, a peak was observed at *q* = 1.2–1.3 nm^−1^, which was also observed in the hydrogel spectra. In the wide-angle region, a hallow was observed in the range *q* = 10–17 nm^−1^, derived from the solvent n-decane. A peak near *q* = 13.5 nm^−1^, overlapping the hallow, and a weak peak near *q* = 15.7 nm^−1^ were also observed. These peaks correspond to interplanar spacings of 0.47 and 0.40 nm, indicating the presence of an angstrom-sized ordered structure. These WAXS peaks were also observed in the hydrogel spectra. The gel fibers in 12-HOA-based organogels are known to consist of two 12-HOA molecules bonded at a 21° angle to the horizontal plane by hydrogen bonding between the carboxyl groups, and the 12-HOA bilayers form a lamellar structure. Therefore, the small angle peak in Figure 2 was assigned to the long spacing of the lamellar structure. In addition, the bilayer in the crystalline gel fiber has a triclinic subcell structure with short spacings of 0.46 and 0.39 nm [4], corresponding to the wide-angle peaks in Figure 2. The SWAXS data for other reported surfactant systems (Figure S2) were similar to those observed for the CTAB system. The above SWAXS results suggest that the hydrogels contain gel fibers with microstructures similar to those found in organogels and surfactant micelles.

The presence of micelles in the hydrogels was indicated by SAXS. For further confirmation, UV-vis measurements were performed on samples admixed with Rhodamine 6G. As shown in Figure S3, the maximum absorption wavelength of Rhodamine 6G in a solvent depended on the solvent polarity, and the maximum absorption wavelengths in water, ethanol, and 1-decanol solutions were 526.2, 531.6, and 535.2 nm, respectively. This shows that the maximum absorption wavelength becomes red-shifted with the decrease in solvent polarity. A red shift also occurs when the surfactant concentration in an aqueous micellar solution comprising Rhodamine 6G increases from below to above the critical micellar concentration (CMC) [32].

**Figure 3:**
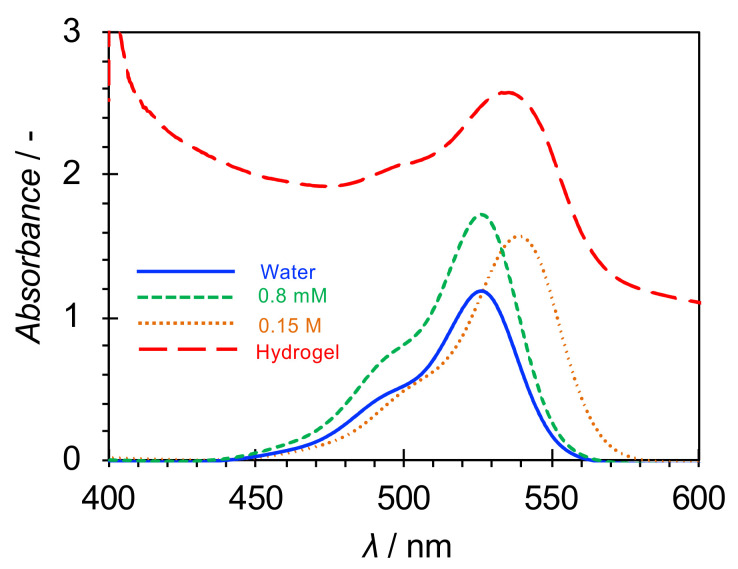
Absorption spectra of Rhodamine 6G in water (1.3 × 10^−5^ M), aqueous surfactant solutions [8 × 10^−4^ and 0.15 M; below and above the critical micellar concentration (CMC), respectively], and hydrogels [hexadecyltrimethylammonium bromide (CTAB); 0.15 M; 12-hydroxyoctadecanoic acid concentration *C*_12-HOA_ = 1.0 wt.%] at 25 °C.

Figure 3 and Figure S4 show the UV-vis spectra of water at 25 °C, surfactant aqueous solutions below and above the CMC, and hydrogels containing Rhodamine 6G. The CMCs of CTAB, CTAC, and CPC in water at 25 °C were determined by the conductivity method (Figure S5), and the results are summarized in Table 2. In addition, the counterion dissociation degree α was determined from the ratio of the two slopes of the conductivity changes below and above the CMC (Table 2).

In all the surfactant systems, the maximum absorption wavelength in the surfactant aqueous solution below the CMC was the same as that of water. On the other hand, the maximum absorption wavelength was red shifted in the surfactant aqueous solution above the CMC (micellar solution). A red shift was also confirmed in the hydrogels, suggesting that micelles were also formed in the hydrogels. However, the degree of red shift in the hydrogels was smaller than that observed in the aqueous surfactant micellar solution. This could be because some of the Rhodamine 6G molecules remain in water due to the interaction of their cationic groups with the carboxyl groups exposed at the surface of the 12-HOA gel fibers.

From the optical microscopic observation and SWAXS and UV-vis results, we concluded that the gel fibers formed in the hydrogels in all the surfactant systems had the same structure as those found in the organogel. In addition, the gel fibers and micelles coexisted in the hydrogels without interfering with each other, indicating that all the hydrogels are orthogonal molecular assembled systems.

### 2.3. Sol-Gel Transition Temperature

**Figure 4:**
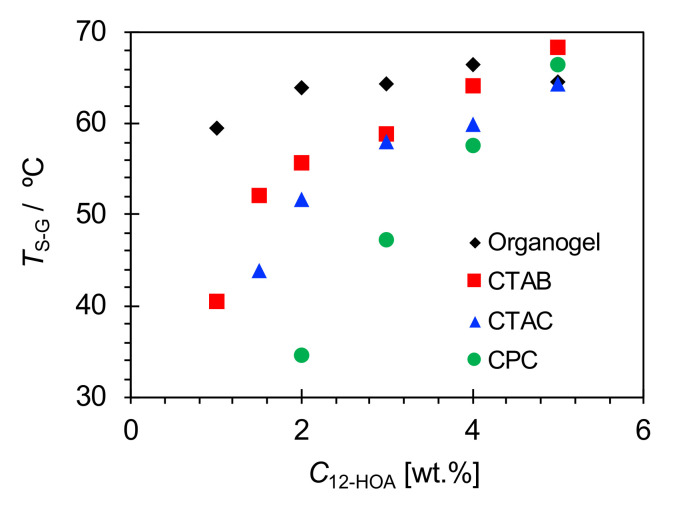
Sol-gel transition temperatures (*T*_S-G_) for the hydrogels and organogels.

Figure 4 shows the results of the *T*_S-G_ measurements with different *C*_12-HOA_ values for the hydrogels and organogels. Notably, at the same C_12-HOA_*,* the *T*_S-G_ of the hydrogel was lower than that of the organogel. In addition, both the organogel and hydrogel *T*_S-G_ values showed gelator-concentration dependences, which were larger in the latter. Moreover, among the hydrogels, the CTAB system exhibited the highest *T*_S-G_. However, this relationship was not held at *C*_12-HOA_ = 5 wt.%, whereby the CTAB and CPC systems presented higher *T*_S-G_ values than that of the organogel, and the CPC system showed a higher *T*_S-G_ than that of the CTAC system. The reason for these inconsistencies at this composition is currently unclear and requires further study.

### 2.4. Viscoelasticity

**Figure 5:**
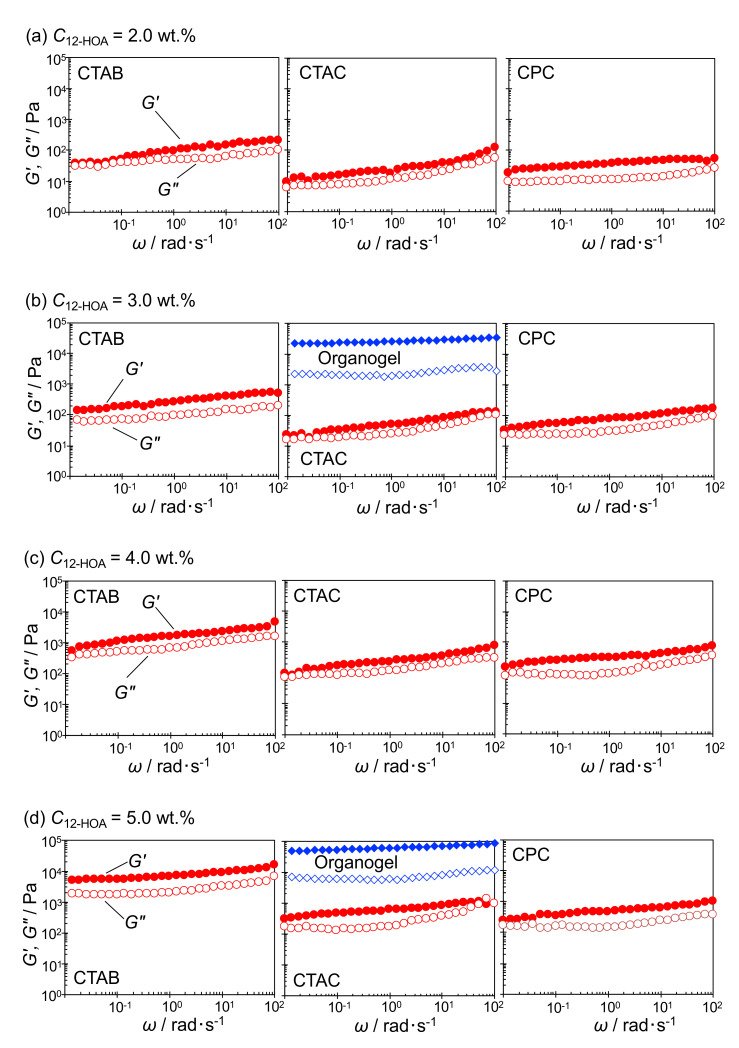
Storage modulus (*G′*, filled symbols) and loss modulus (*G″*, open symbols) as functions of the oscillatory shear frequency (*ω*) of the hydrogels (12-hydroxyoctadecanoic acid concentration *C*_12-HOA_ = 2.0–5.0 wt.%) in the hexadecyltrimethylammonium bromide (CTAB; left), hexadecyltrimethylammonium chloride (CTAC; middle), and hexadecylpyridinium chloride (CPC; right) systems at different *C*_12-HOA_ (2.0–5.0 wt.%) and of the organogels (n-decane/12-HOA, *C*_12-HOA_ = 3.0 and 5.0 wt.%) at 25 °C. (**a**) *C*_12-HOA_ = 2.0 wt.%, (**b**) *C*_12-HOA_ = 3.0 wt.%, (**c**) *C*_12-HOA_ = 4.0 wt.% and (**d**) *C*_12-HOA_ = 5.0 wt.%.

Figure 5 shows the dynamic viscoelasticity of the hydrogels and organogels at 25 °C. In all the results, the storage modulus *G’* is greater than the loss modulus *G”*, regardless of the angular velocity *ω*, indicating gel or solid properties. In addition, *G’* and *G”* increased with increasing gelator concentration. The hydrogel *G’* and *G”* values were lower than those of the organogels and showed a larger *ω* dependence. This suggests that hydrogels are weaker gels [33] than organogels. Considering the differences among the surfactant systems, *G’* and *G”* showed the largest values in the CTAB system.

**Figure 6:**
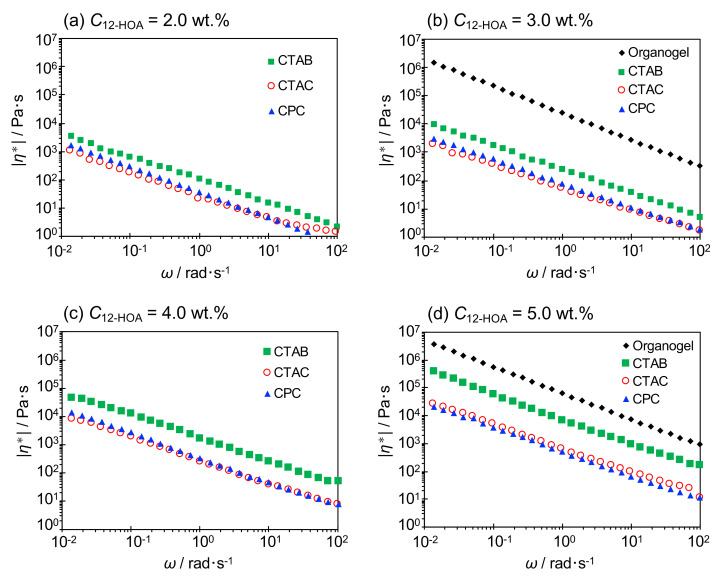
Absolute complex viscosity values of the hydrogels and organogel (n-decane/12-hydroxyoctadecanoic acid (12-HOA)) as a function of the oscillatory shear frequency (*ω*) at 25 °C. (**a**) *C*_12-HOA_ = 2.0 wt.%, (**b**) *C*_12-HOA_ = 3.0 wt.%, (**c**) *C*_12-HOA_ = 4.0 wt.% and (**d**) *C*_12-HOA_ = 5.0 wt.%.

The absolute value of the complex viscosity |η*| was obtained from *G’* and *G”* by Equation (1), and the results are shown in Figure 6.

(1) $$\left|\eta^{*}\right|=\frac{\sqrt{G{’}^{2}+G{’’}^{2}}}{\omega }$$

In all the systems, |η*| decreased with increasing *ω*, indicating that the network structure in the gels was destroyed by strain. Moreover, |η*| was the largest in the organogels and, among the hydrogels, in the CTAB system. In addition, |η*| increased with increasing gelator concentration in the hydrogels.

## 3. Discussion

The hydrogels formed by the SMG method showed higher cgc and lower *T*_S-G_ and viscoelasticity values than those of the organogels. The hydrogel cgc values were much higher than that of the organogel obtained from 12-HOA and n-dodecane, which is approximately 0.2 wt.% [34]. The cgc increases with increasing solvent polarity and hydrogen-bonding ability of the solvent [13]; therefore, it seems reasonable that the cgc of a hydrogel is larger than that of the organogel. On the other hand, a more significant difference in the hydrogels obtained by the SMG method, compared to organogels, is the presence of surfactant micelles in the gels. Thus, part of the 12-HOA molecules remained solubilized in the micelles and did not contribute to gel fiber formation, leading to the increase in cgc.

According to the Gibbs–Thomson effect, the melting point of a crystalline particle decreases significantly with increasing curvature, implying that the melting point of thinner gel fibers should be lower than thicker ones. However, from the optical microscopy data in Figure 1, there is no significant difference between the organogel and hydrogel fiber thicknesses. Hence, it was necessary for us to consider other factors. The organogel and hydrogel *T*_S-G_ values decreased with decreasing gelator concentration, as shown in Figure 4. Therefore, the solubilization effect can also explain the lower *T*_S-G_ values of the hydrogels over those of the organogels. This is because the actual amount of gelator molecules forming gel fibers in the hydrogels is lower than that in the organogels, even at the same C_12-HOA_. Furthermore, focusing on the degree of gelator concentration dependence on the *T*_S-G_, the maximum difference in *T*_S-G_ among the organogels at different gelator concentrations was approximately 5 °C, while the differences among the hydrogels in a given surfactant system were much greater (more than 20 °C). When the gelator concentration decreases under constant surfactant concentration, the ratio of the gelator solubilized in the micelle to the gelator concentration in the whole system increases. This results in a more pronounced solubilization effect of the gelator on the *T*_S-G_. An additional factor that lowers the *T*_S-G_ in hydrogels is the difference in the polarity of the solvent in which the gelator molecules are dissolved. When considering the sol-gel transition of the gels stabilized by SAFiNs, upon increasing the temperature the gel fibers need to be dissolved in a solvent. Gel fibers in the 12-HOA-based organogels were simply dissolved in the solvent used. On the other hand, gel fibers in the 12-HOA-based hydrogels cannot be dissolved in water because of their poor solubility; however, they can be solubilized in surfactant micelles. Since 12-HOA is amphiphilic, it is reasonable that the molecule should be located at the palisade layer of the surfactant micelle. In addition, the carbon number in the hydrophobic chain was C18 for 12-HOA and C16 for the cationic surfactants employed in this study. Therefore, the environment in which 12-HOA is solubilized is polar to some extent. The *T*_S-G_ of 12-HOA-based organogels is lower in polar than in nonpolar solvents [12]. Therefore, the *T*_S-G_ is lower in hydrogels than in organogels, owing to the difference in the polarity of n-decane and the micelle cores.

This lower viscoelasticity in hydrogels over that in organogels can also be explained by the solubilization effect of the gelator, because the number or amount of gel fibers should decrease by the part of the gelator remaining in the micelles. In fact, the CTAB hydrogel at *C*_12-HOA_ = 5.0 wt.% and organogel at *C*_12-HOA_ = 3.0 wt.% have similar *G’*, *G”*, and |η*| levels, reflecting the contribution of the gelator solubilizing effect to the decrease in viscoelasticity.

On comparing the cgc values of the hydrogels in the different surfactant systems, the cgc in the CTAB system was the lowest, suggesting that gelator solubilization was the lowest in this system. Conversely, the *T*_S-G_ and viscoelasticity were the highest in the CTAB system because the solubilization effect of the gelator in the micelles is the lowest, and larger amounts of gel fibers are formed. The difference in the solubilization effect, depending on the surfactant, was explained as follows: since gel fibers are in the crystalline state, it is reasonable to consider that the solubilization of the gelator after gel formation is in a non-equilibrium state. It is understood that the mechanisms of gel fiber formation in a 12-HOA organogel depend on the cooling rate during gel preparation. Crystal growth mainly occurs by molecular diffusion of the gelator to the crystal growth surface when cooling at rates less than 5 °C min^−1^ [35]. For hydrogel preparation by the SMG method, the cooling rate around the *T*_S-G_ approximates 4 °C min^−1^ [16]; therefore, in this study, gel fiber formation was dependent on the diffusion rate of the 12-HOA molecules. In organogel systems, the 12-HOA molecules can freely diffuse in the solvent during the gel fiber formation process, while in the hydrogel forming system, the gelator molecules are stuck in the micelles and diffuse together with the micelles. Therefore, 12-HOA crystal nuclei are formed within one micelle at an early stage of the gel fiber formation process, and gelator molecules solubilized in other micelles must be supplied to the crystal nuclei for crystal growth to proceed. Hence, the other micelles need to approach a micelle comprising a nucleus by self-diffusion to transfer the gelator molecules between the micelles or merge the micelles together. In either case, the diffusion rate and collision frequency of the micelles control the process. The micelle radii of each surfactant at 25 °C were reported to be 3.55 nm for CTAB [36], 2.72 nm for CTAC [36], and 1.81 nm for CPC [37]. Considering the Stokes–Einstein equation, the CTAB micelles display the smallest diffusion coefficient. In addition, as shown in Table 2, the degree of counter ion dissociation of the CTAB micelles is smaller than that of the other cationic surfactant micelles, indicating that the electrostatic repulsion between the micelles is weaker than that of the other surfactant systems. Therefore, micelle collision and fusion occur more easily and frequently, and, in the gel fiber formation process, the gelator molecules are the most efficiently supplied to the crystal nuclei in the CTAB system; this results in the smallest solubilization effect in this system.

Finally, we discuss the difference of the SMG hydrogels from other 12-HOA-based hydrogels. In this study, we demonstrated that gel fibers with the same morphology and microstructure as the 12-HOA organogel were formed in the SMG hydrogel through optical microscopy and SWAXS. Hydrogelation using 12-HOA was reported in the systems containing 12-HOA with the presence of alkanolamines [17,18] or alkylamines [19,20] which form ionic complexes with 12-HOA. Therefore, in the alkanolamine systems, rod-shaped fibers with small aspect ratios are formed. In addition, the fiber morphology changes to a hollow cylinder instead of the helical ribbon shape formed in the 12-HOA organogel. In the alkylamine system, the gel network structure is no longer uniform SAFiNs but spherulites are dispersed while the helical ribbon shape is maintained. The most characteristic feature of the SMG method is that SAFiNs in the 12-HOA organogel can be formed in water. This could lead to the technology to assemble supramolecular aggregates composed of water-insoluble functional supramolecular aggregates into aqueous systems without impairing their functionality.

## 4. Materials and Methods

### 4.1. Materials

12-HOA (99%) was purchased from Sigma-Aldrich (St. Luis, MO, USA); the product was identified as an R-enantiomer [14]. CTAB (98.0%) and CPC (98.0%) were purchased from Tokyo Chemical Industry Co. (Tokyo, Japan). CTAC (95.0%) was purchased from Kanto Chemical Co. (Tokyo, Japan), while Rhodamine 6G was purchased from Tokyo Chemical Industry Co. Deionized water prepared by Elix 3 (Nihon Millipore, Tokyo, Japan) was used to prepare the samples.

### 4.2. Sample Preparation

All compounds were weighed in screw-capped glass tubes. For homogeneous mixing, they were heated to ≈90 °C in an aluminum block heater, followed by cooling in air to room temperature (25–30 °C). The samples were maintained in an incubator set at 25 °C for at least 24 h prior to measurement. The gelator concentration C_12-HOA_ was defined as the percentage weight of 12-HOA in the system.

### 4.3. Gelation Test and Sol-Gel Transition Temperatures

A sample was determined as being in the gel or sol state by the inverted test tube method. In this method, a test tube containing the sample was placed upside down. If the sample did not flow after 30 min, the sample was determined to be gelled. The sol-gel transition temperature (*T*_S-G_) was also determined by the inverted test tube method performed in a temperature-controlled water bath. The temperature was gradually increased until the sample flowed.

### 4.4. Optical Microscopy

A differential interference contrast microscope, model BX51 (Olympus, Tokyo, Japan) with a CCD camera, model DP22 (Olympus, Tokyo, Japan) was used. The samples were placed between untreated glass plates and observed at room temperature (20–25 °C).

### 4.5. Differential Scanning Calorimetry (DSC)

A DSC 6200 instrument (Seiko Instruments, Chiba, Japan) was used. The samples were placed in aluminum pans (Hitachi High-Tech Science, Tokyo, Japan) and sealed tightly. The DSC curves were recorded at a heating rate of 5 °C min^−1^.

### 4.6. Rheological Measurements

An AR-G2 rheometer (TA Instruments, New Castle, DE, USA) was used. All measurements were performed using a plate-plate geometry (diameter of moving plate, 4.0 cm). The bottom plate temperature was controlled using a Peltier unit. Initially, oscillation-strain sweeps were performed with a constant oscillation frequency of 1 Hz to identify the linear viscoelastic (LVE) range of each sample. Subsequently, oscillation-frequency sweeps (0.01–100 rad s^−1^) were performed at a constant strain (0.070–0.15%, depending on samples) within the LVE range.

### 4.7. SWAXS Measurements

SWAXS measurements were performed using a SAXSess camera (Anton Paar, Graz, Austria) attached to a PW3830 sealed-tube anode X-ray generator (PANalytical, Almelo, The Netherlands) operated at 40 kV and 50 mA. A monochromatic X-ray beam of Cu–Kα radiation (*λ* = 0.1542 nm) with a focused line-shape was generated using a Göbel mirror fitted with a block collimator. The thermostatic sample holder unit (TCS 120, Anton Paar, Graz, Austria) was part of the SAXSess system and provided a temperature accuracy of ±0.1 °C. Two-dimensional (2D) scattering patterns were first recorded on an image plate (IP) detector (Cyclone, Perkin Elmer, Waltham, MA, USA) and were subsequently integrated into 1D scattering intensities, *I*(*q*), as a function of the absolute value of the scattering vector, *q*, using SAXSQuant software (Anton Paar, Graz, Austria).

### 4.8. Ultraviolet-Visible (UV-vis) Spectroscopy

UV-vis spectra were recorded on a V-730 spectrophotometer (Jasco, Tokyo, Japan). A 10 mm thick quartz sample cell was used. The sample temperature was controlled by a temperature unit specially designed for the instrument. The concentration of Rhodamine 6G was fixed at 1.3 × 10^−5^ M in all the samples. Gelled samples were dispersed in water using a vortex mixer to achieve sufficient transmittance.

## 5. Conclusions

The SMG method for hydrogel formation using an organogelator, 12-HOA, was performed using three cationic surfactants, CTAB, CTAC, and CPC having different hydrophilic groups. Hydrogel formation, structural analysis, and mechanical property evaluation were performed on the hydrogels. In each system, the hydrogels were constructed using a macroscopic and microscopic fiber-like structure similar to that of 12-HOA-based organogels. In addition, the coexistence of gel fibers and surfactant micelles was confirmed in all the hydrogels by UV-vis spectroscopy and X-ray scattering. Among the different surfactant systems, the CTAB system had the lowest minimum gelation concentrations and highest sol-gel transition temperatures and viscoelasticity. This study showed that the SMG method, already reported for the CTAB system [16], is also feasible in other cationic surfactant systems. In addition, the hydrophilic group of the surfactant affects the gel fiber formation, which could lead to better understanding of the formation and stabilization mechanisms of SAFiNs gels. In the future, we need to expand on anionic or nonionic surfactants and other organogelators to establish the universality of the SMG method.

## Figures and Tables

**Table 1:** Gelation test in the water/surfactant/12-hydroxyoctadecanoic acid (12-HOA) systems.

	C_12-HOA_ [wt.%]									
Surfactant ^1^	0.5	0.75	1.0	1.25	1.5	1.75	2.0	3.0	4.0	5.0
CTAB	sol	sol	gel	-	gel	-	gel	gel	gel	gel
CTAC	sol	-	sol	sol	sol	gel	gel	gel	gel	gel
CPC	sol	-	sol	-	sol	sol	gel	gel	gel	gel

^1^ Surfactant concentration in the system was fixed at 0.15 M. Definitions: CTAB = hexadecyltrimethylammonium bromide, CTAC = hexadecyltrimethylammonium chloride, and CPC = hexadecylpyridinium chloride.

**Table 2:** Critical micellar concentration (CMC) and counterion dissociation degree (α).

	CMC/mM	α/-
CTAB	0.91	0.28
CTAC	1.32	0.42
CPC	0.99	0.49

Definitions: CTAB = hexadecyltrimethylammonium bromide, CTAC = hexadecyltrimethylammonium chloride, and CPC = hexadecylpyridinium chloride.

## Supplementary Materials

Supplementary materials can be found at https://www.mdpi.com/1422-0067/21/21/8046/s1.

## Abbreviations

LMG	low-molecular-weight gelator
SAFiNs	self-assembled fibrillar networks
12-HOA	12-hydroxyoctadecanoic acid
BME	bicontinuous microemulsion
Lα	lamellar liquid crystal
SMG	surfactant-mediated gelation
CTAB	hexadecyltrimethylammonium bromide
CTAC	hexadecyltrimethylammonium chloride
CPC	hexadecylpyridinium chloride
cgc	critical gelation concentration
SWAXS	small- and wide-angle X-ray scattering
CMC	critical micellar concentration
DSC	differential scanning calorimetry
LVE	linear viscoelastic

## References

[1] Weiss R.G., Terech P. (2006). Molecular Gels. Materials with Self-Assembled Fibrillar Networks.

[2] Caran K.L., Lee D.-C., Weiss R.G., Liu X.Y., Li J.-L. (2013). Molecular Gels and their Fibrillar Networks. Soft Fibrillar Materials: Fabrication and Applications.

[3] Tachibana T., Kambara H. (1969). Studies of Helical Aggregates of Molecules. I. Enantiomorphism in the Helical Aggregates of Optically Active 12-Hydroxystearic Acid and Its Lithium Salt. Bull. Chem. Soc. Jpn..

[4] Tachibana T., Mori T., Hori K. (1980). Chiral Mesophase of 12-Hydroxyoctadecanoic Acid in Jelly and in the Solid State. I. A New Type of Lyotropic Mesophase in Jelly with Organic Solvents. Bull. Chem. Soc. Jpn..

[5] Tachibana T., Mori T., Hori K. (1981). Chiral Mesophases of 12-Hydroxyoctadecanoic Acid in Jelly and in the Solid State. II. A New Type of Mesomorphic Solid State. Bull. Chem. Soc. Jpn..

[6] Sato H., Hori K., Sakurai T., Yamagishi A. (2008). Long Distance Chiral Transfer in a Gel: Experimental and Ab Initio Analyses of Vibrational Circular Dichroism Spectra of R- and S-12-Hydroxyoctadecanoic Acid Gels. Chem. Phys. Lett..

[7] Sakurai T., Masuda Y., Sato H., Yamagishi A., Kawaji H., Atake T., Hori K. (2010). A Comparative Study on Chiral and Racemic 12-Hydroxyoctadecanoic Acids in the Solutions and Aggregation States: Does the Racemic Form Really Form a Gel?. Bull. Chem. Soc. Jpn..

[8] Terech P. (1991). Small-Angle-Scattering Study of 12-Hydroxystearic Physical Organogels and Lubricating Greases. Colloid Polym. Sci..

[9] Terech P. (1992). 12-D-Hydroxyoctadecanoic Acid Organogels: A Small Angle Neutron Scattering Study. J. Phys. II France.

[10] Terech P., Rodriguez V., Barnes J.D., McKenna G.B. (1994). Organogels and Aerogels of Racemic and Chiral 12-Hydroxyoctadecanoic Acid. Langmuir.

[11] Terech P., Pasquier D., Bordas V., Rossat C. (2000). Rheological Properties and Structural Correlations in Molecular Organogels. Langmuir.

[12] Wu S., Gao J., Emge T.J., Rogers M.A. (2013). Solvent-Induced Polymorphic Nanoscale Transitions for 12-Hydroxyoctadecanoic Acid Molecular Gels. Cryst. Growth Des..

[13] Gao J., Wu S., Emge T.J., Rogers M.A. (2013). Nanoscale and Microscale Structural Changes Alter the Critical Gelator Concentration of Self-assembled Fibrillar Networks. Cryst. Eng. Comm..

[14] Laupheimer M., Preisig N., Stubenrauch C. (2015). The Molecular Organogel *n*-Decane/12-Hydroxyoctadecanoic Acid: Sol-Gel Transition, Rheology, and Microstructure. Colloids. Surf. A.

[15] Koitani S., Dieterich S., Preisig N., Aramaki K., Stubenrauch C. (2017). Gelling Lamellar Phases of the Binary System Water−Didodecyldimethylammonium Bromide with an Organogelator. Langmuir.

[16] Aramaki K., Koitani S., Takimoto E., Kondo M., Stubenrauch C. (2019). Hydrogelation with a Water-Insoluble Organogelator–Surfactant Mediated Gelation (SMG). Soft Matter.

[17] Douliez J.-P., Gaillard C., Navailles L., Nallet F. (2006). Novel Lipid System Forming Hollow Microtubes at High Yields and Concentration. Langmuir.

[18] Fameau A.-L., Cousin F., Navailles L., Nallet F., Boué F., Douliez J.-P. (2011). Multiscale Structural Characterizations of Fatty Acid Multilayered Tubes with a Temperature-Tunable Diameter. J. Phys. Chem. B.

[19] Nakagawa M., Kawai T. (2018). Chirality-Controlled Syntheses of Double-Helical Au Nanowires. J. Am. Chem. Soc..

[20] Nakagawa M., Kawai T. (2019). Tuning Gel Sol Transition Behavior of a Hydrogel Based on 12-Hydroxystearic Acid and a Long-Chain Amidoamine Derivative. Bull. Chem. Soc. Jpn..

[21] Fameau A.-L., Rogers M.A. (2020). The Curious Case of 12-Hydroxystearic Acid—The Dr. Jekyll & Mr. Hyde of Molecular Gelators. Curr. Opin. Colloid Interface Sci..

[22] Stubenrauch C., Tessendorf R., Strey R., Lynch I., Dawson K.A. (2007). Gelled Polymerizable Microemulsions. 1. Phase Behavior. Langmuir.

[23] Stubenrauch C., Tessendorf R., Salvati A., Topgaard D., Sottmann T., Strey R., Lynch I. (2008). Gelled Polymerizable Microemulsions. 2. Microstructure. Langmuir.

[24] Magno M., Tessendorf R., Medronho B., da Graça Martins Miguel M., Stubenrauch C. (2009). Gelled Polymerizable Microemulsions. Part 3 Rheology. Soft Matter.

[25] Laupheimer M., Jovic K., Antunes F.E., da Graça Martins Miguel M., Stubenrauch C. (2013). Studying Orthogonal Self-Assembled Systems: Phase Behaviour and Rheology of Gelled Microemulsions. Soft Matter.

[26] Laupheimer M., Sottmann T., Schweins R., Stubenrauch C. (2014). Studying Orthogonal Self-Assembled Systems: Microstructure of Gelled Bicontinuous Microemulsions. Soft Matter.

[27] Xu Y., Laupheimer M., Preisig N., Sottmann T., Schmidt C., Stubenrauch C. (2015). Gelled Lyotropic Liquid Crystals. Langmuir.

[28] Peng K., Sottmann T., Stubenrauch C. (2019). Gelled Non-Toxic Microemulsions: Phase Behavior & Rheology. Soft Matter.

[29] Steck K., Schmidt C., Stubenrauch C. (2018). The Twofold Role of 12-Hydroxyoctadecanoic Acid (12-HOA) in a Ternary Water—Surfactant—12-HOA System: Gelator and Co-Surfactant. Gels.

[30] Boekhoven J., Brizard A.M., Stuart M.C.A., Florusse L., Raffy G., Del Guerzo A., van Esch J.H. (2016). Bio-Inspired Supramolecular Materials by Orthogonal Self-Assembly of Hydrogelators and Phospholipids. Chem. Sci..

[31] Stubenrauch C., Gießelmann F. (2016). Gelled Complex Fluids: Combining Unique Structures with Mechanical Stability. Angew. Chem. Int. Ed..

[32] Carey M.C., Small D.M. (1969). Micellar Properties of Dihydroxy and Trihydroxy Bile Salts: Effects of Counterion and Temperature. J. Colloid Interface Sci..

[33] Ross-Murphy S.B., Shatwell K.P. (1993). Polysaccharide Strong and Weak Gels. Biorheology.

[34] Dieterich S., Sottmann T., Giesselmann F. (2019). Gelation of Lyotropic Liquid-Crystal Phases–The Interplay between Liquid Crystalline Order and Physical Gel Formation. Langmuir.

[35] Rogers M.A., Marangoni A.G. (2009). Solvent-Modulated Nucleation and Crystallization Kinetics of 12-Hydroxystearic Acid: A Nonisothermal Approach. Langmuir.

[36] Matzinger S., Hussey D.M., Fayer M.D. (1998). Fluorescent Probe Solubilization in the Headgroup and Core Regions of Micelles: Fluorescence Lifetime and Orientational Relaxation Measurements. J. Phys. Chem. B.

[37] Gunaseelan K., Dev S., Ismail K. (2000). Estimation of Micellization Parameters of Cetylpyridinium Chloride in Water Using the Mixed Electrolyte Model for Conductance. Indian J. Chem. A.

