# Supplementary material for: Effect of the Cationic Head Group on Cationic Surfactant-Based Surfactant Mediated Gelation (SMG)
Source: Int J Mol Sci. 2020 Oct 28;21(21):8046. doi: 10.3390/ijms21218046 (PMC7663335; doi:10.3390/ijms21218046)
Supplement: Supplementary file 1 [file ijms-21-08046-s001.pdf]

International Journal of
Molecular Sciences

MDPI

Supplementary Materials

Effect of Cationic Head Group on Cationic Surfactant-Based Surfactant Mediated Gelation (SMG)

Kenji Aramaki [1,*], Eriko Takimoto [2] and Takumi Yamaguchi [2]

[1] Graduate School of Environment and Information Sciences, Yokohama National University; aramaki-kenji-cr@ynu.ac.jp
* Correspondence: aramaki-kenji-cr@ynu.ac.jp; Tel.: +81-45-339-4300

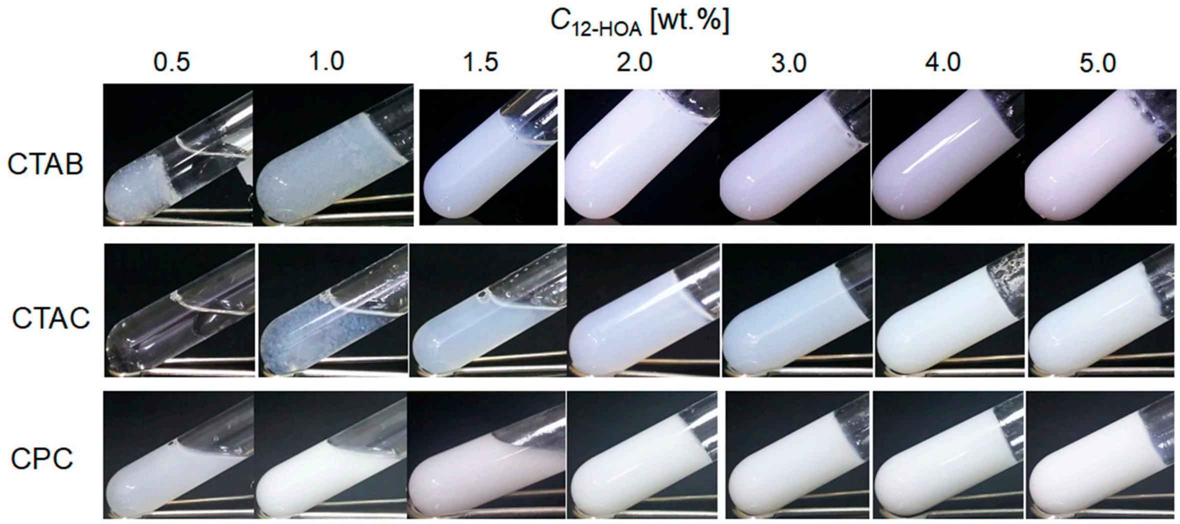

Figure S1 Photos of the hydrogel samples in the water/surfactant/12-hydroxyoctadecanoic acid (12-HOA) systems at different 12-HOA concentrations. Surfactant concentration in the system was fixed at 0.15 M. Flowable and non-flowable samples are indicated as "sol" and "gel" in Table 1, respectively. Note that the sample in the CTAB system at $C_{12\text{-HOA}}$ = 1.0 wt.% was seemed as non-flowable, however we judged as a "sol" sample because it was easily flowable with a slight shock.

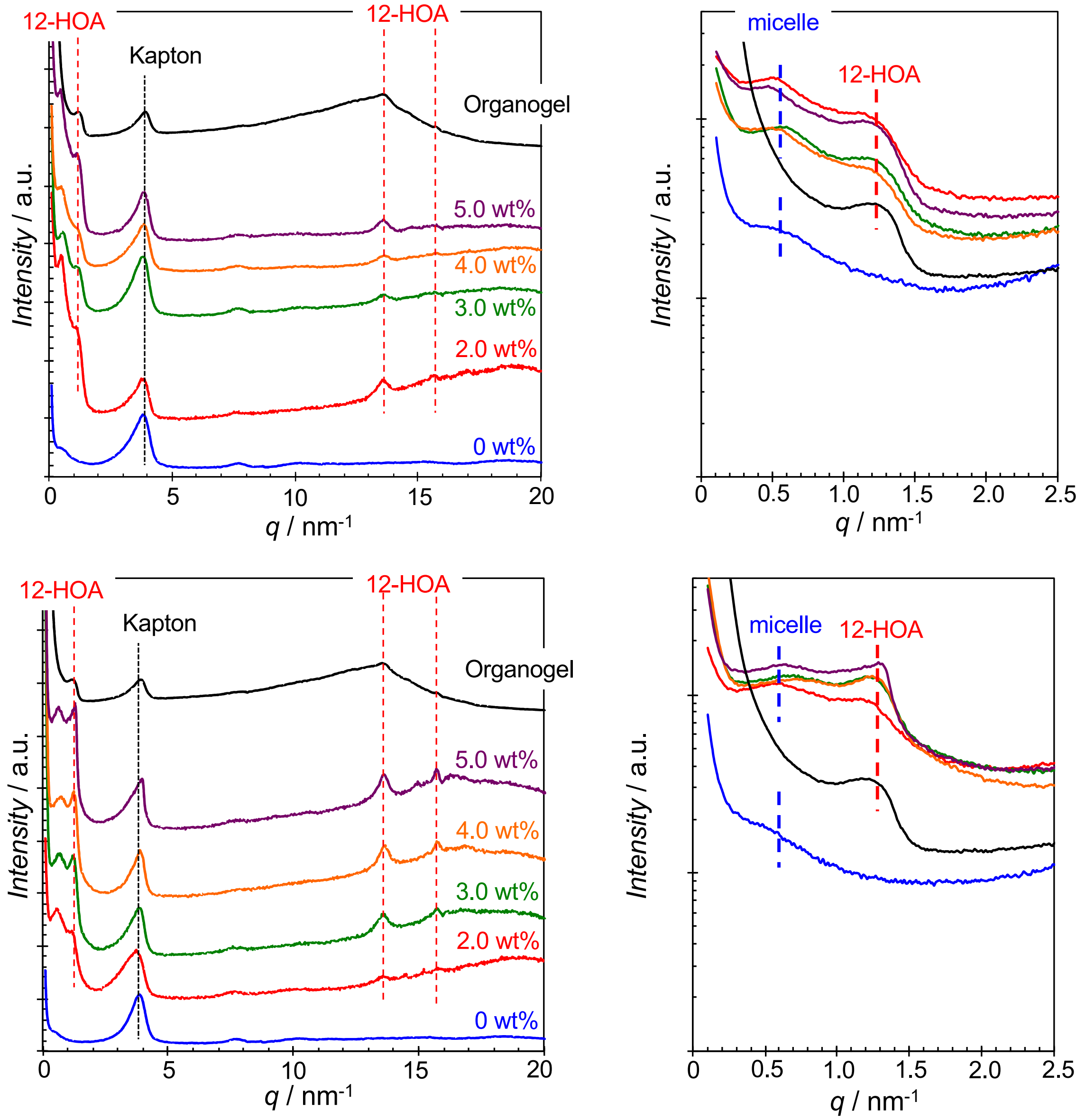

Figure S2 SAXS and WAXS curves of the hydrogels in the CTAC (upper) and CPC (lower) systems, the nongelled surfactant micellar solutions and the organogels (n-decane/12-HOA, $C_{12\text{-HOA}}$ = 3.0 wt.%) at 25 °C. The small angle peaks at around q = 4 nm^{-1} are from Kapton window.

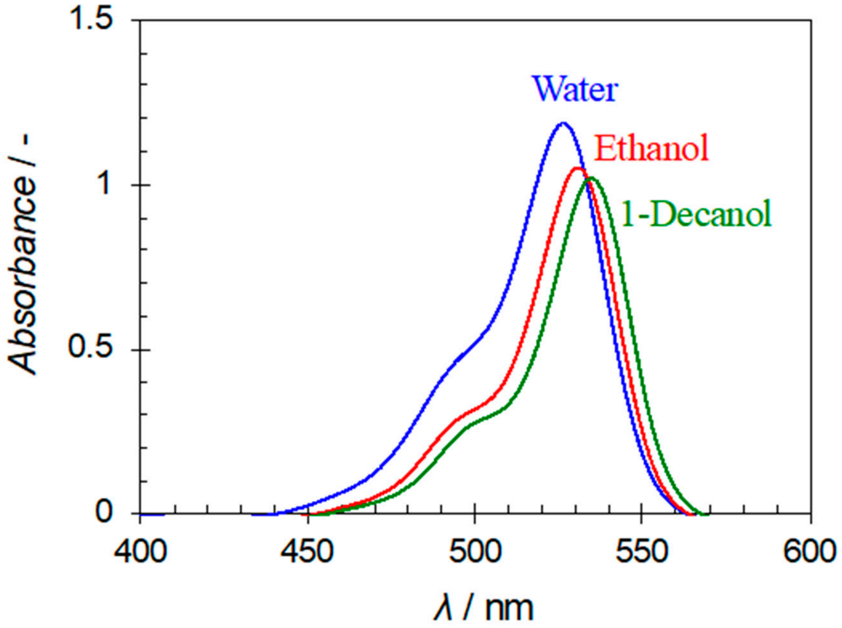

Figure S3 UV-vis spectra of rhodamine 6G in different solvent at 25 °C

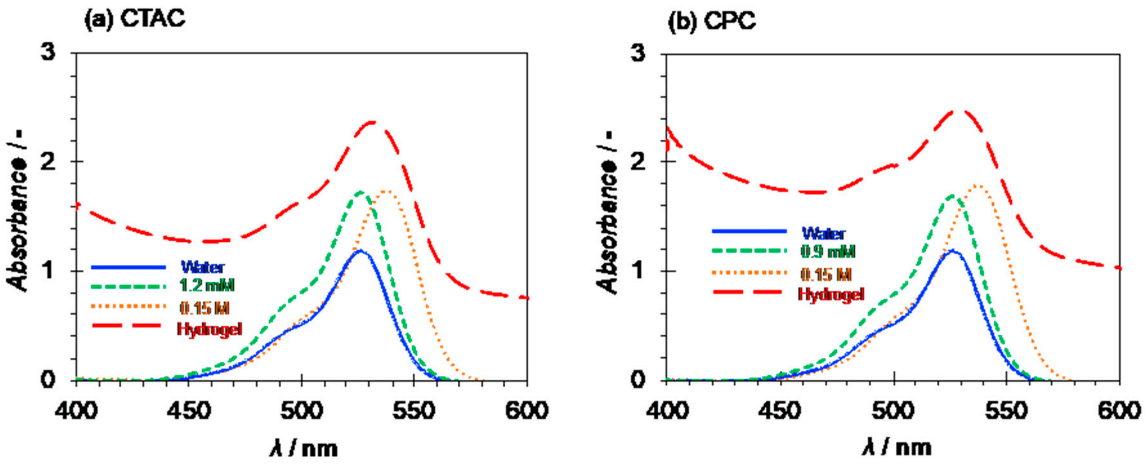

Figure S4 (a) Absorption spectra of rhodamine 6G in water (1.3×10^{-5} M), in aqueous CTAC solutions (1.2×10^{-3} and 0.15 M, below and above CMC, respectively) and in the hydrogel (CTAC, 0.15 M; $C_{12\text{-HOA}}$ = 1.5 wt.%) at 25 °C. (b) Absorption spectra of rhodamine 6G in water (1.3×10^{-5} M)), in aqueous CPC solutions (9×10^{-4} and 0.15 M, below and above CMC, respectively) and in the hydrogel (CPC, 0.15 M; $C_{12\text{-HOA}}$ = 2.0 wt.%)) at 25 °C.

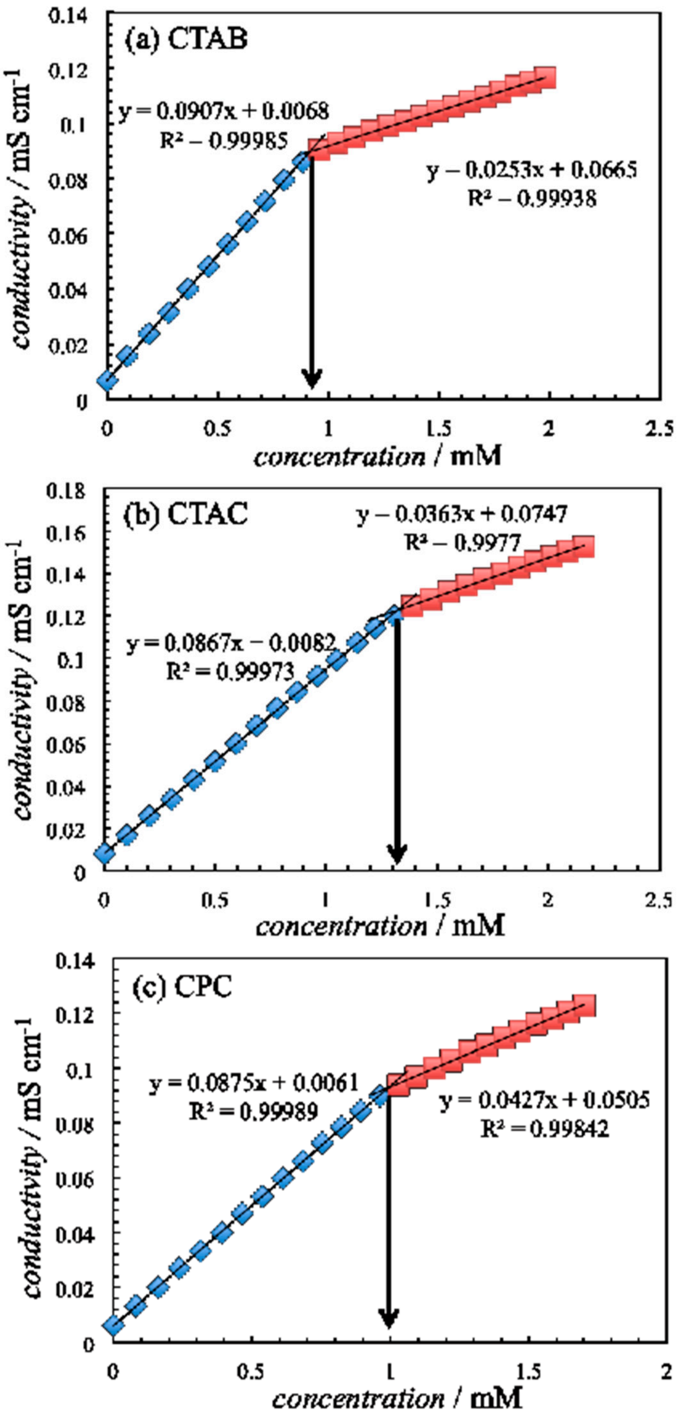

Figure S5 Specific conductivity is plotted against surfactant concentration at 25 °C (a) CTAB, (b) CTAC, (c) CPC
